# The GSK3β-β-catenin-TCF1 pathway improves naive T cell activation in old adults by upregulating miR-181a

**DOI:** 10.1038/s41514-021-00056-9

**Published:** 2021-02-08

**Authors:** Zhongde Ye, Timothy M. Gould, Huimin Zhang, Jun Jin, Cornelia M. Weyand, Jörg J. Goronzy

**Affiliations:** 1 From the Department of Medicine, Division of Immunology and Rheumatology, Stanford University, Stanford, CA 94305 USA; 2 Department of Medicine, Veterans Affairs Palo Alto Health Care System, Palo Alto, CA 94306 USA

**Keywords:** Transcription, Cell signalling

## Abstract

MicroRNAs play an important role in the regulation of T cell development, activation, and differentiation. One of the most abundant microRNAs in lymphocytes is miR-181a, which controls T cell receptor (TCR) activation thresholds in thymic selection as well as in peripheral T cell responses. We previously found that miR-181a levels decline in T cells in the elderly. In this study, we identified TCF1 as a transcriptional regulator of pri-miR-181a. A decline in TCF1 levels in old individuals accounted for the reduced miR-181a expression impairing TCR signaling. Inhibition of GSK3ß restored expression of miR-181a by inducing TCF1 in T cells from old adults. GSK3ß inhibition enhanced TCR signaling to increase downstream expression of activation markers and production of IL-2. The effect involved the upregulation of miR-181a and the inhibition of DUSP6 expression. Thus, inhibition of GSK3ß can restore responses of old T cells by inducing miR-181a expression through TCF1.

## Introduction

The dramatic increase in life expectancy in the 21st century is an evident success of modern medicine. However, with the substantial increase in human life span, new concerns about healthy aging have emerged. Immunity declines during aging, as shown by the increased susceptibility to infection by both previously encountered as well as new pathogens and by the decreased efficacy of vaccination^1–3^. Aging-related diseases, such as cancer, cardiovascular and neurodegenerative diseases, are becoming a world-wide challenge and new strategies to prevent these diseases will have a major impact on improving healthy aging. In the elderly, the benefits of vaccination to prevent infectious disease are limited, which contribute to their increased morbidity, mainly because of the adaptive immune system’s inability to generate protective immunity. Each year, the greatest numbers of such deaths are associated with influenza viruses. For underlying respiratory and circulatory deaths, 90% of influenza-associated deaths occurred among persons aged 65 years or older^4–8^. Recently, the current SARS-CoV-2 pandemic is becoming a global health threat. The higher mortality rate in the elderly caused by COVID-19 may be at least in part due to the lack of an adaptive immune response, which has become a challenge for biomedical aging research^9–12^. Therefore, understanding the underlying age-associated defects of adaptive immunity in humans is imperative.

Human responses to infectious diseases or vaccinations depend on many different cell populations and the interactions among them. For example, T lymphocytes are composed of naive, memory, and effector cells with highly variable clonal sizes and vast repertoires of TCRs. The elderly immune system has to cope with a decreased ability to produce new T cells and failure to maintain homeostasis in these cellular systems. However, emerging data suggest that homeostatic mechanisms are robust enough to maintain a large and diverse CD4 TCR repertoire with age. The notable compartment shrinkage and clonal expansions were seen in naive CD8 T cells^13–16^. In addition to population aspects, identification of potentially targetable cellular defects is receiving strong interest^17,18^.

The discovery of microRNA (miRNA) genes revealed an unexpected layer of genetic programming that regulates different aspects of vertebrate biological processes, such as hematopoietic lineage differentiation and immune response^19,20^. Age-associated changes in miRNA may ultimately contribute to the failure in T cell homeostasis exemplified by the loss in naïve cells^21^. MicroRNA genes encode primary RNA transcripts (pri-miRNA), which are sequentially processed into mature miRNAs. These 22-nt RNAs can repress the expression of protein-coding genes by targeting cognate messenger RNAs for degradation or translational repression^22,23^. Our previous observations indicate that miR-181a levels decline during T cell aging and its target signaling pathways are thus rendered defective due to the upregulation of negative regulatory loops^24^. Naive CD4 T cells from the elderly have reduced extracellular signal-related kinase (ERK) phosphorylation upon TCR activation. Defects in ERK signaling are caused by several phosphatases including dual specific phosphatase 6 (DUSP6), whose protein levels increase with age due to the decline in miR-181a-mediated inhibition^24^. Modification of TCR signaling by miR-181a also involves other negative feedback molecules, including PTPN22, SHP2, DUSP5 and SIRT1^25,26^ Conditional knock-out mice with miR-181a^-/-^ T cells exhibit features of human immune aging including reduced expansion of antigen-specific T cells in anti-viral responses, delayed viral clearance and a defect in the generation of tissue-residing memory T cells^27^.

Functional improvements of the aged adaptive immune systems may be accomplished by inhibiting miR-181a targets or ultimately by restoring miR-181a expression. Here, we aimed at identifying gene-regulatory factors that influence miR-181a expression during T cell aging. In previous studies, we have described a putative pri-miR-181a enhancer, near position 198,904,300 on chromosome 1^28^. Here we show that this enhancer is regulated by a transcription factor complex including YY1 and T cell factor 1 (TCF1), both of which decline in naive CD4 T cells with age. TCF1 levels could be increased by inhibiting glycogen synthase kinase 3ß (GSK3ß) in T cells from old individuals, thereby restoring miR-181a expression and consequently improving TCR sensitivity to stimulation. This finding implicates the potential use of small molecules to boost immune responses in the elderly.

## Results

### TCF1 regulates pri-miR-181a/b1 expression

**Fig. 1:**
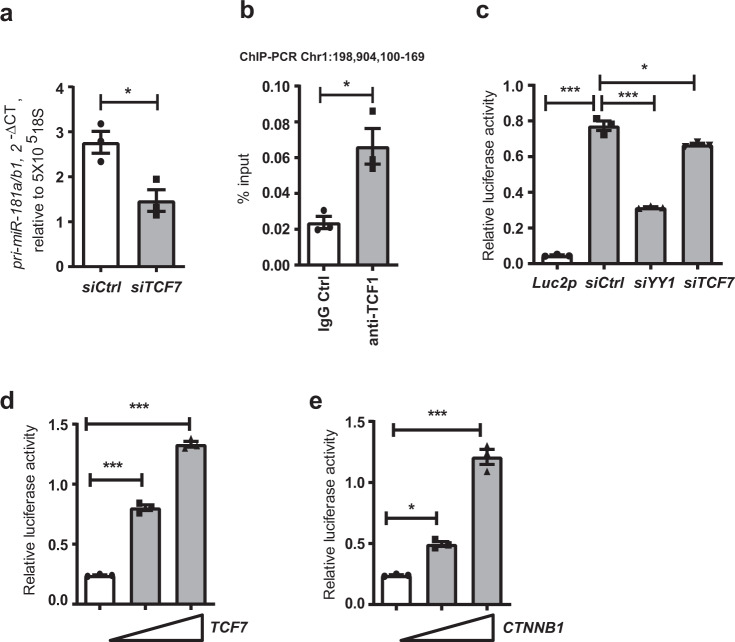
Regulation of pri-miR-181a/b1 expression by TCF1. **a** Naive CD4 T cells from young adults were transfected with *TCF7* siRNA or control siRNA and assayed for pri-miR-181a/b1 expression after 48 h by q-PCR. Data are shown as mean±SEM (*n* = 3). **b** ChIP assay was performed on naïve CD4 T cells with anti-human TCF1 antibodies, and the sequence representing a previously identified pri-miR-181a/b1 enhancer region (chr1:198,904,100-169) was amplified. Precipitation with normal IgG was used as control. Data are shown as mean±SEM (*n* = 3). **c** Sequences corresponding to pri-miR-181a/b1 enhancer region (chr1:198,904,065-558) were cloned into a pGL4.27 [luc2P/minP/Hygro] plasmid. Dual-luciferase reporter assays were performed in HEK293T cells transfected with *TCF7* siRNA, *YY1* siRNA, or control siRNA. Data are shown as mean±SEM (n = 3). **d**, **e** Increasing amounts (0 ng, 30 ng or 100 ng) of *TCF7* (d) and *CTNNB1* (e) –containing plasmids were co-transfected with the pri-miR-181a/b1-enhancer-Luc2p reporter construct into HEK293T cells. Reporter activities after 48 h are shown as mean±SEM (n = 3). Comparisons were done by two-tailed paired *t* test in **a**, **b**; or, by one-way ANOVA with post-hoc Tukey test in **c**, **d**, and **e**. Significance levels are indicated as *P < 0.05, **P < 0.005, ***P < 0.0001.

In our previous study, we found that pri-miR-181a/b1 declines with age, and this decline is at least in part regulated by the transcription factor YY1^28^. To identify additional transcription factors that could be therapeutically targeted, we used PROMO and TRANSFAC to predict binding motifs within the pri-miR-181a/b1 enhancer region and identified a TCF1/LEF1 binding motif. To confirm that TCF1 is a potential candidate that may regulate the expression of pri-miR181a/b1, human naive CD4 T cells were transfected with small interfering RNA (siRNA) specific for TCF1, or non-specific siRNA as a control. Knockdown of *TCF7* (encoding TCF1) effectively reduced the expression of pri-miR-181a/b1 (Fig. 1a). In ChIP-PCR assays of naive CD4 T cells from young adults, we found an enrichment of pri-miR-181a/b1 enhancer sequences in the precipitates with anti-TCF1 antibodies compared to control IgG (Fig. 1b). In addition, silencing *TCF7* reduced the pri-miR-181a/b1 enhancer activity compared to transfection with control scrambled siRNA as measured by reporter gene assays in HEK293T cells (Fig. 1c). Conversely, overexpression of TCF1 or co-activator β-catenin increased activity of the pri-miR-181a/b1 enhancer reporter in a dose-dependent manner (Fig. 1d, e). Taken together, we conclude that TCF1 is a direct regulator of pri-miR-181a/b1 expression.

### Inducing TCF1 restores miR-181a expression in old adults

**Fig. 2:**
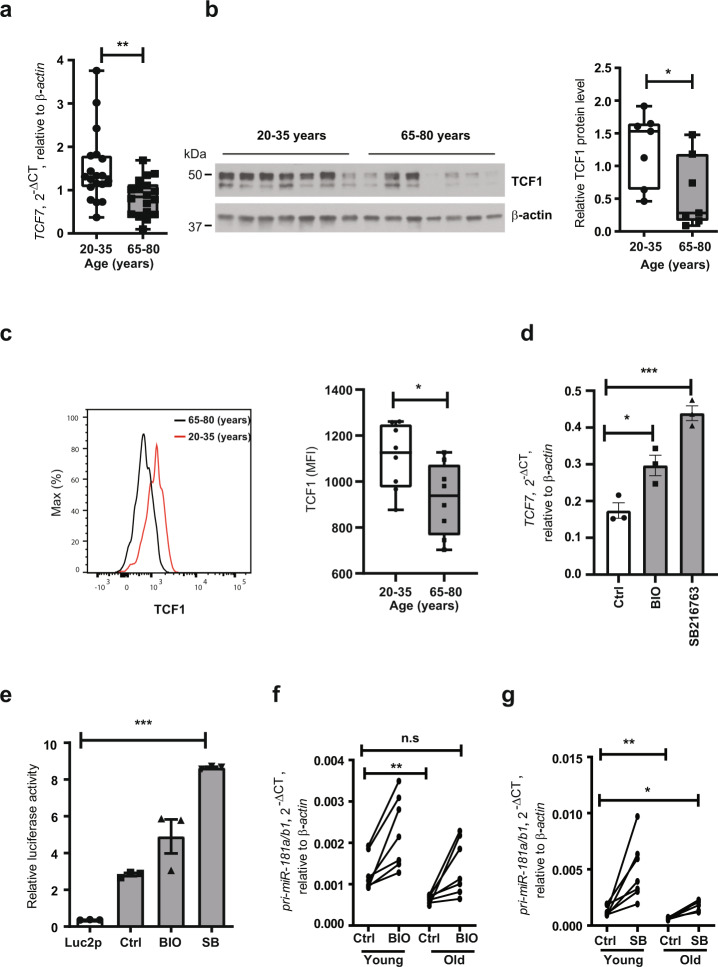
Restoration of miR-181a expression in old naive CD4 T cells by inducing TCF1 activity. **a** *TCF7* transcripts in naive CD3 + CD4 + CD45RO- T cells were quantified by qPCR. Results for 20–35 (*n* = 19) and 65–85 (*n* = 18) year-old individuals are shown as box plots of *TCF7* transcripts relative to *ACTB*. **b** TCF1 protein levels in CD3 + CD4 + CD45RO- naive T cells from seven young and seven old adults are shown as Western blots (left) and box plots of relative densities (right). Uncropped Western blots in Supplementary Figure 4. All samples in the blot derive from the same experiment and were processed in parallel. **c** TCF1 expression levels in naive T cells from eight young and eight old adults are shown as representative histograms (left) and summary box plots (right). **d** Naive CD4 T cells from old individuals were treated with GSK3β inhibitors 6-bromoindirubin-3-oxime (BIO) or SB216763 for 48 h. *TCF7* transcripts quantified by qPCR are shown as mean±SEM (*n* = 3). **e** BIO (1 µM) or SB216763 (5 µM) were added to HEK293T cells transfected with pri-miR-181a enhancer reporter constructs 12 h after transfection. Reporter activities after 48 h are shown as mean±SEM (n = 3). **f**, **g** Naive CD4 T cells from seven young and seven old individuals were cultured with DMSO solvent control or BIO (**f**, 1 µM) or SB216763 (g, 5 µM) for 48 h. Pri-miR-181a/b1 transcripts were quantified by qPCR. Comparisons were done by two-tailed unpaired *t* test in **a**, **b**, **c**, **f**, and **g**; or by one-way ANOVA with post-hoc Tukey test in **d**, **e**. Significance levels are indicated as *P < 0.05, **P < 0.005, ***P < 0.0001.

The expression of pri-miR-181a/b1 decreases with age in naïve CD4 T cells^28^. Since TCF1 can positively regulate the expression of pri-miR-181a/b1, we next examined the expression level of TCF1 in naïve CD4 T cells of young (*n* = 19 and elderly (*n* = 18) individuals. As shown in Fig. 2a and b, both TCF1 transcript and protein levels were significantly decreased in T cells from older adults. Reduced TCF1 expression levels in naïve CD4 T cells from the elderly were confirmed by flow cytometry (Fig. 2c, gating strategy in Supplementary Figure 1). Importantly, the entire population was shifted, documenting that the reduced TCF1 expression was a feature of the entire population and not a contamination with a subset such as TEMRAs. Hence, we reasoned that inducing TCF1 expression would restore miR-181a expression in the elderly. Inhibiting GSK3ß is known to enhance β-catenin/TCF1 signaling to its target genes^29^. Inhibition of GSK3ß with two different inhibitors, BIO and SB216763, increased expression of *TCF7* transcripts in resting naïve CD4 T cells (Fig. 2d). In addition, BIO and SB216763 increased the pri-miR-181a/b1 enhancer activity as measured by reporter gene assays in HEK293T cells (Fig. 2e).

Expression of miR-181a is decreased in aged T cells, resulting in defective T cell signaling in the elderly^24^. Therefore, we hypothesized that restoring the expression of miR-181a would enhance T cell function and thus improve the immune response in the elderly. Since inhibiting GSK3ß activity up-regulated expression of TCF1 in resting naive CD4 T cells, we further evaluated the expression levels of pri-miR-181a/b1 in naïve CD4 T cells from young and elderly individuals treated with GSK3ß inhibitors, BIO or SB216763. Irrespective of age, inhibition of GSK3ß increased expression of pri-miR-181a/b1; in naïve CD4 T cells from elderly individuals, both inhibitors restored expression nearly to a level comparable to that in young individuals not treated with GSK-3ß inhibitors (Fig. 2f, g). This effect was confirmed for naive CD4 T cells purified by cell sorting (Supplementary Fig. 2a, b). miR-181a expression is lower in memory than naive cells and a further age-dependent decline is seen in a subset of older adults. Inhibition of GSK3ß also increased expression of pri-miR-181a/b1 in memory cells, however without reaching the level seen in naïve CD4 T cells (Supplementary Fig. 2 c, d).

### Inhibiting GSK3β enhances TCR signaling in the elderly

**Fig. 3:**
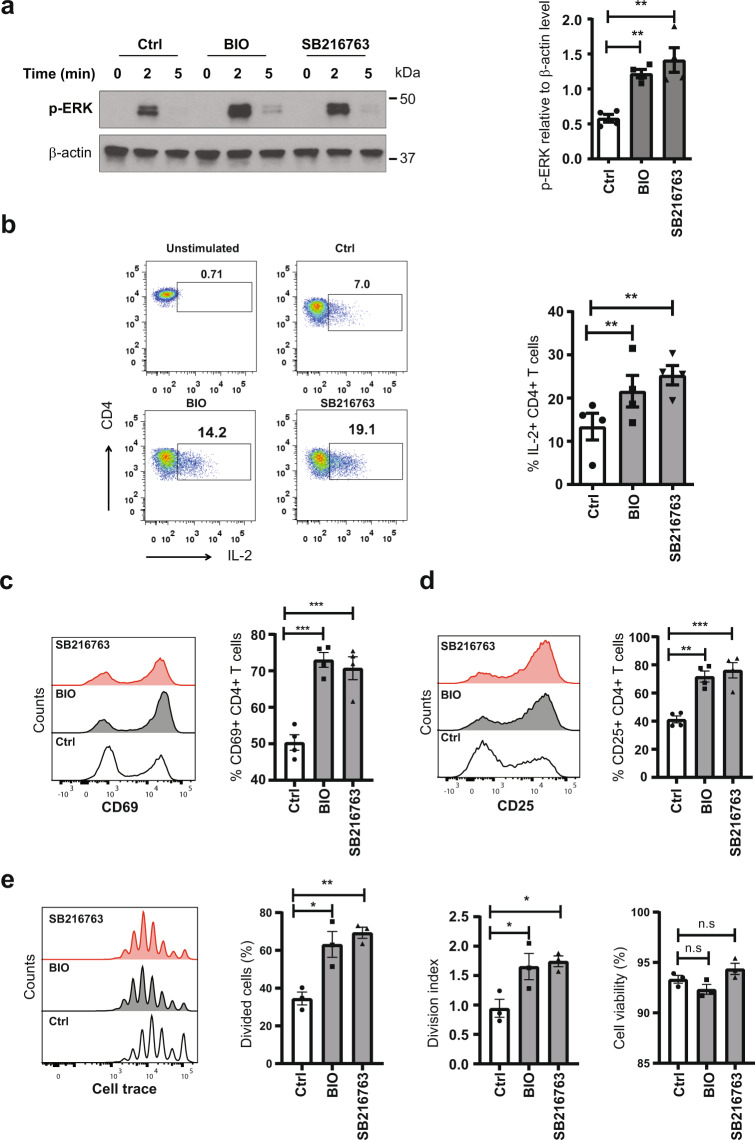
GSK3β inhibition enhances TCR signaling in T cells from old individuals. **a** Naive CD4 T cells from old adults were pre-treated with DMSO solvent control or GSK3β inhibitors BIO (1 µM) or SB216763 (5 µM) for 48 h and then stimulated by CD3/CD28 cross-linking. Representative immunoblots of pERK (Thr202/Tyr204) at indicated time points (left) and mean±SEM of pERK relative to β-actin levels at 2 min (*n* = 4, right) are shown. Uncropped Western blots in Supplementary Fig. 4. All blots derive from the same experiment and were processed in parallel. **b** Naive CD4 T cells from old adults were pre-treated with 1 µM BIO or 5 µM SB216763 for 48 h; cells were washed once in PBS, seeded into 48-well plates, and stimulated with Dynabeads at a bead-to-cell ratio of 1:2 for 24 h, and GolgiPlug was added for the last 8 h. Intracellular cytokine staining for IL-2 production is shown as representative scatter plots (left) and frequencies of IL-2-producing cells are shown as mean±SEM (*n* = 4, right). **c**–**e** Naive CD4 T cells from old adults were pre-treated with 1 µM BIO or 5 µM SB216763 and cultured for 24 h (CD69), 48 h (CD25), or 5 days (proliferation) in plates coated with CD3/CD28 antibodies. **c** CD69 expression is shown as representative histograms (left) and frequencies of CD69 are shown as mean±SEM (*n* = 4, right). **d** CD25 expression is shown as representative histograms (left) and frequencies of CD25 are shown as mean±SEM (*n* = 4, right). **e** Cell proliferation is shown as representative histograms (left); cell proliferation index and cell viability are shown as mean±SEM (*n* = 3, right). Comparisons were done by one-way ANOVA with post-hoc Tukey test. Significance levels are indicated as *P < 0.05, **P < 0.005, ***P < 0.0001.

Decreased expression of miR-181a impairs the sensitivity of TCR signaling^24,28^. Since GSK3ß inhibition restored expression of pri-miR-181a/b1 in naive CD4 T cells from elderly individuals, we further tested whether such inhibition would modulate the sensitivity to TCR stimulation. We treated naive CD4 T cells from elderly individuals with GSK3ß inhibitors BIO or SB216763 for 48 h and examined TCR signaling at different time points upon CD3/CD28 cross-linking. As shown in Fig. 3a, inhibition of GSK3ß activity increased ERK phosphorylation downstream of TCR activation. To analyze the functional consequences of GSK3ß inhibition in the elderly, we examined production of interleukin 2 (IL-2) by flow cytometry and measured IL-2 levels in the presence and absence of GSK3ß inhibitors after TCR activation. Figure 3b shows that inhibiting GSK3ß increased IL-2 production in naive CD4 T cells from elderly individuals. Further, we evaluated CD69 and CD25 expression in activated naive CD4 T cells from the elderly treated with GSK3ß inhibitors. Figures 3c and d show that inhibition of GSK3ß increased CD69 and CD25 expression. Cells pretreated with GSK3ß inhibitors also increased cell division but did not significantly affect T cell survival after 5 days in culture (Fig. 3e). Thus, inhibiting GSK3ß activity increases the sensitivity to TCR stimulation and activation.

### Regulation of TCR signaling by GSK3β inhibitors is dependent on miR-181a

**Fig. 4:**
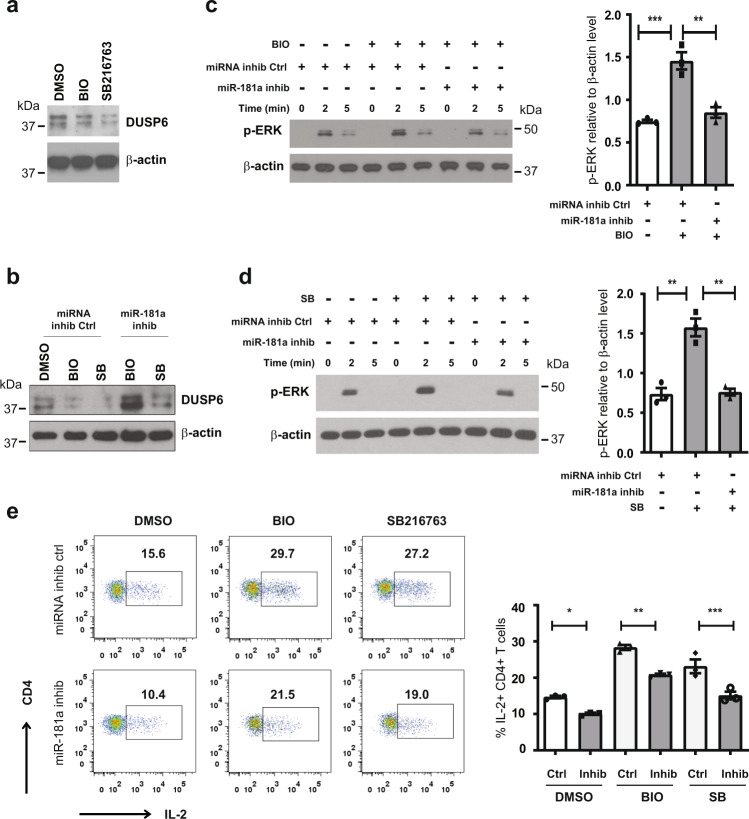
GSK3β inhibitors regulate T cell receptor signaling and T cell activation through controlling miR-181a expression. **a** Naive CD4 T cells were treated with GSK3β inhibitors BIO (1 µM) or SB 216763 (5 µM) for 48 h. Cell lysates were subjected to Western blotting for the miR-181a target DUSP6. β-actin was used as loading control. Data are representative of two experiments. **b** Naive CD4 T cells were transfected with miR-181a inhibitor or miRNA inhibitor negative control and then cultured and analyzed for the expression of DUSP6 after culture with GSK3ß inhibitors as described in (**a**). Data are representative of two experiments. **c**, **d** Naive CD4 T cells, transfected with miR-181a inhibitor or miRNA inhibitor negative control, were treated with GSK3β inhibitors BIO (c, 1 µM) or SB 216763 (d, 5 µM) as described above. Immunoblots of pERK signals (Thr202/Tyr204) at indicated times after CD3/CD28 cross-linking (left) and mean±SEM of pERK relative to β-actin levels at 2 min (*n* = 3, right) are shown. Uncropped Western blots in Supplementary Fig. 4. All samples in a blot derive from the same experiment and were processed in parallel. **e** Naive CD4 T cells, transfected with miR-181a inhibitor or miRNA inhibitor negative control, were pre-treated with DMSO, 1 µM BIO, or 5 µM SB216763 as described above. Intracellular cytokine staining for IL-2 is shown as representative scatter plots (left) and mean frequencies±SEM of IL-2-producing cells of three experiments (right). Comparisons were done by one-way ANOVA with post-hoc Tukey test. Significance levels are indicated as *P < 0.05, **P < 0.005, ***P < 0.0001.

**Fig. 5:**
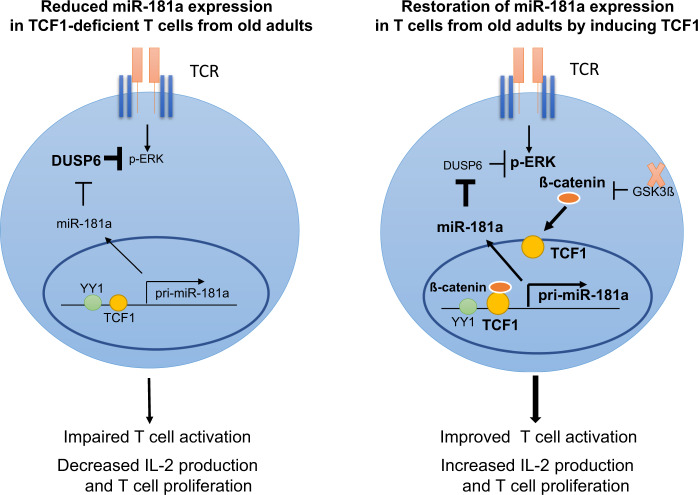
Model of TCF1-mediated regulation of miR-181a dependent on GSK3ß. T cell aging is associated with a loss of miR-181a expression due to reduced TCF1 expression (left). Reduced miR-181a expression contributes to impaired T cell function by activating negative regulatory signaling pathways by miR-181a targets, e.g., ERK dephosphorylation by DUSP6. Inhibition of GSK3ß increases the expression of TCF1 and its target miR-181a in T cells from old adults, thus enhancing downstream T cell activation and proliferation (right).

As previously identified, miR-181a regulates TCR signaling through one of its target genes, DUSP6^24^. Therefore, we examined DUSP6 levels in naive CD4 T cells from the elderly after treatment with GSK3ß inhibitors BIO or SB216763. As shown in Fig. 4a, inhibition of GSK3ß activity by either inhibitor decreased DUSP6 protein levels; pre-treating cells with a miR-181a inhibitor abrogated this decrease consistent with the notion that the decrease in DUSP6 is mediated by upregulation of miR-181a (Fig. 4b). Since DUSP6 selectively dephosphorylates ERK, we evaluated ERK phosphorylation in naive CD4 T cells from the elderly treated with GSK3ß inhibitors. To determine the involvement of miR-181a upregulation, we also transfected the cells with a miR-181a antagonist or miRNA inhibitor negative control. As shown in Fig. 4c and d, transfecting cells with miR-181a inhibitor abrogated the increased T cell signaling produced by the GSK3ß inhibitors. Transfection with the miR-181a inhibitor reduced IL-2 production after TCR activation, consistent with the notion that miR-181a, even at the reduced levels in the elderly CD4 T cells, is still functionally important. GSK3ß inhibition increased the production of IL-2; this upregulation was at least in part reversible by transfected miR-181a antagonist (Fig. 4e). Taken together, these results suggest that T cell activation in older individuals can be modulated by increasing miR-181a expression downstream of GSK3ß inhibition (Fig. 5).

## Discussion

The abundance of miR-181a in T cells influences their responsiveness to activation signals. Expression of miR-181a is dynamically regulated during T cell development with higher expression in immature T cells and lower expression in more differentiated T cell populations^26^. Most striking are age-associated declines in miR-181a expression in naive and to a lesser extent in memory T cells that may account for some of the age-associated defects in T cell function (Fig. 2 and Supplementary Fig. 2). Expression of miR-181a is transcriptionally controlled with pri-miR-181a/b1 transcripts declining with age^24,28^. In the current study, we demonstrate that TCF1 is a direct regulator of a previously described pri-miR-181a/b1 enhancer region^28^. Moreover, we show that the decline in miR-181a expression mirrors that of decreased TCF1 levels for aged naive CD4 T cells, and that miR-181a expression can be restored by inducing TCF1 expression through the inhibition of GSK3ß activity. One immediate implication is the potential use of small molecule GSK3ß inhibitors as a strategy to boost T cell responses in the elderly.

Initial studies on miR-181a emphasized its role as an intrinsic regulator of TCR signaling thresholds by targeting several phosphatases that function as negative regulators of key signaling molecules including CD3ζ and ERK^26^. The high expression of miR-181a in thymocytes has been implicated in facilitating positive T cell selection when low-affinity recognition of self-antigens provides survival signals. Conversely, lack of miR-181a expression prevented the deletion of T cells that are strongly reactive toward positively selecting peptides^30^. Equally important, miR-181a expression is required for the development of Tregs and iNKT cells^31,32^.

Consistent with miR-181a regulating expression of various phosphatases in thymic development, the decline of miR-181a with age correlated with increased protein expression of DUSP6 and reduced ERK phosphorylation upon TCR stimulation. In our functional read-out systems, we, therefore, focused on TCR-induced phosphorylation events and the transcription of IL-2 that in naive T cells is a sensitive marker of AP-1 activation downstream of pERK. Consistently, we found that inducing TCF1, or stabilizing the TCF1 co-activator β-catenin through GSK3ß inhibition, decreased DUSP6 levels and potentiated ERK phosphorylation; the effects were largely abolished by a miR-181a inhibitor, implicating an upregulation of pri-miR-181a/b transcription. T cell activation markers CD69 and CD25 expression levels were also increased by inhibition of GSK3ß as was cell proliferation.

We and others have shown that the consequences of miR-181a deficiency for T cell responses are broader than the lowering of the TCR activation threshold by a selected set of phosphatases^25,33^. Similar to old individuals, infected mice with miR-181a-deficient T cells had a delay in viral clearance due to impaired generation of antigen-specific short-lived CD8 effector T cells, while expansion of antigen-specific CD4 T cells was increased in a compensatory manner^27^. This defect was not susceptible to DUSP6 inhibition, and preliminary studies indicate a role for another miR-181a target, SIRT1. Restoring pri-miR-181a transcription would be a more promising intervention to improve T cell function, rather than inhibiting one of the miR-181a targets.

In previous studies, we have found YY1 to be an important regulator of pri-miR-181a/b1 transcription. YY1 expression in T cells declines with age, in particular in CD8 T cells where age-associated losses in chromatin accessibility are enriched for YY1 binding motifs^34,35^. However, it is currently unknown how to target the defect in YY1 binding. Here we showed that TCF1 binds to the same pri-miR-181a/b1 enhancer region as YY1 and, more importantly, increasing TCF1 activity upregulated pri-miR-181a/b transcripts irrespective of YY1.

TCF1 was observed as essential for T cell development in the thymus soon after its discovery^36,37^. NOTCH signaling in early thymocyte precursor cells induces the transcription of *TCF7* encoding TCF1 that confers T cell lineage commitment, in part through the induction of GATA3^36^. In fibroblasts, TCF1 erases repression marks and activates T cell-restricted genes^38^. Throughout T cell development, TCF1 is highly enriched at thousands of regulatory elements that become accessible at the earliest stage and persist until T cell maturation. MicroRNA-181a is one of the most highly expressed microRNAs in thymocytes and is transiently upregulated at the late double-negative to double-positive stages in T cell development^39^. Given our data here and the close temporal relationship of TCF1 and miR-181a in T cell development, TCF1 may in part affect T cell developmental processes through the regulation of miR-181a expression.

TCF1 is an effector transcription factor in the WNT signaling pathway; the long form of TCF1 directly associates with ß-catenin^40^, an important component of the canonical WNT signaling pathway. The expression of ß-catenin is strictly regulated by the degradation complex composed of adenomatous polyposis coli (APC), axis inhibition protein (AXIN), GSK3ß and casein kinase 1 (CK1). Inhibition of GSK3ß can dephosphorylate and stabilize ß-catenin. Stabilized ß-catenin translocates into the nucleus and competes with TCF repressor proteins such as Groucho, thereby initiating TCF-mediated transcription, including the induction of TCF1 itself^41^. In our study, overexpression of TCF1, as well as ß-catenin, activated the pri-miR-181a/b1 enhancer, suggesting that TCF1-dependent miR-181a expression is upregulated through the WNT pathway. Accordingly, increased TCR signaling due to GSK3β inhibition can be at least partially reversed by a miR-181a antagonist.

Given the broad role of TCF1 in T cell biology, its reduced expression with age is likely to have consequences independent of its effect on miR-181a expression. In searching for TCF1 targets that could contribute to the effect on T cell activation^42^, *TCF7* decreased *PIM1* transcription, while inhibition of GSK3ß decreased its expression. Moreover, PIM1 expression is increased with age, consistent with reduced TCF1 expression^43^. Accordingly, PIM1 inhibition increased activation-induced CD69 and CD25 expression (Supplementary Fig. 3). Thus, the beneficial effect of GSK3β inhibition on T cell activation also involves TCF1 targets other than miR-181a to affect T cell signaling, however, our rescue experiments proofed that miR-181 was one major pathway.

Independent of TCR signaling and TCR threshold calibration, the reduced expression of TCF1 is likely to have effects on T cell differentiation and function, in part mediated through miR-181a, in part through other molecules^42,44^. Kared and colleagues found loss of TCF1 with age especially in T stem cell memory cells^45^, and activation of WNT/TCF1 signaling by inhibiting GSK3ß was found to maintain pluripotency in human and mouse embryonic stem cells^46^. Further, the GSK3ß inhibitor 6-bromoindirubin 3′-oxime (BIO) elevated the proportion of naive T cells and delayed T cell differentiation during homeostatic T cell expansion in lympho-depleted mice by activating WNT signaling^47^. Thus, the reduced expression of TCF1 observed with age may be associated with a failure to maintain quiescence. Indeed, epigenetic signatures of differentiation have been found in naive T cells with increasing age, indicating a loss in stemness. However, such a failure in maintaining quiescence is unlikely related to the loss in miR-181a expression, which on the contrary would increase the threshold for T cell activation.

Similar to homeostatic proliferation, TCF1 in T cell differentiations drives gene-regulatory pathways that promote stem-like or memory fates^48–50^. Downregulation of TCF1 by IL-12 after T cell activation favors differentiation to short-lived effector T cells^51^. In contrast, WNT signaling during antigen-activation is important for the generation of stem-like memory cells with TCF1 promoting EOMES expression. Under condition of chronic infection, TCF1 confers resistance to T cell exhaustion in a subset of T cells^48,50^. For CD4 T cells, TCF1 induces BCL6 expression, thereby biasing T cell differentiation towards T_FH_ and memory cell differentiation. Importantly, aged naive CD4 T cells preferentially differentiate into short-lived effector T cells at the expense of T_FH_ and memory T cells^52^. Taken together, the aging T cell system exhibit several features that are consistent with TCF1 dysfunction. Obviously, not all of them are related to reduced miR-181a expression. However, it is of interest to note that mice with miR-181a-deficient T cells have a reduced ability to generate tissue-residing antigen-specific T cells after viral infection and exhibit less effective memory responses^27^.

In summary, here we identified that pri-miR-181a/b1 is a direct target of TCF1 and that loss of miR-181a with age is at least in part explained by loss of TCF1. Restored expression of miR-181a in aged naive CD4 T cells could be accomplished by inhibiting GSK3ß, thus suggesting a novel strategy to improve immune responses in the elderly (Fig. 5).

## Methods

### Study population and cell purification

Fifty-three (*n* = 53) young individuals aged 20–35 years and sixty-eight (*n* = 68) old individuals aged 65–85 years were included in the study. All individuals in the study population were healthy, meaning subjects had no evidence of acute illness, current or previous history of immune-mediated diseases, cancer (excluding limited basal cell carcinoma), or any chronic disease not controlled by oral medications. All study participants gave written informed consent, and the study protocol was approved by the Stanford University Institutional Review Board. Peripheral blood mononuclear cells (PBMCs) were separated from whole blood by density centrifugation using Ficoll media (Lymphoprep^TM^, STEMCELL Technologies). Naive CD4 T cells were purified by either cell sorting gated on CD4 + CD45RA + CD62L + from pre-purified T cells, or from PBMC by negative selection with human CD4 T cell enrichment cocktail (15062, STEMCELL Technologies) followed by negative selection using anti-human CD45RO microbeads (130- 046-001, Miltenyi Biotec), or from PBMC with negative selection by using Easysep^TM^ human Naive CD4 + T cell isolation kit (19555, STEMCELL Technologies). Subset purity monitored by FACS routinely exceeded 95%.

### Antibodies and chemicals

Antibodies specific for TCF1 (#2203 S) and pERK (T202/Y204, #4377 S) from Cell Signaling Technology, and DUSP6 (ab76310) from Abcam, were used at a dilution of 1 in 1000 for immunoblotting or 1 in 50 for ChIP-PCR. Antibodies specific for β-actin were from Santa Cruz Biotechnology (sc-47778 HRP) and used at 1 in 10000. Fluorochrome-conjugated antibodies were from BD Biosciences including CD4-APC (555349), CD69- PerCP-Cy5.5 (560738) and CD45RA-PE-Cy™7 and BioLegend including CD62L-PE (304806), CD25-APC (302610) and human IL-2 Alexa-Fluor-488 (500314). All antibodies for flow cytometry were used at 1 in 50. PIM1 inhibitor (526521), GSK3β inhibitors, BIO (2’Z,3’E)-6-Bromoindirubin-3ʹ-oxime (361550) and SB216763 (S3442), were from Sigma.

### Reporter luciferase reporter assays

The pri-miR-181a/b1 enhancer region was cloned into the pGL4.27 Luc2p/minP vector (Promega) as described^28^. Thirty nanograms of pri-miR-181a/b1 Luc2p/Peak 1 was co-transfected with 1 ng Renilla luciferase reporter pRL-TK (Promega) into HEK293T cells, or Luc2p empty vector (negative control). For TCF1 loss-of-function on pri-miR-181a/b1 enhancer activity, *TCF7* siRNA (Dharmacon A-019735-13-0005) or non-targeting siRNA (Dharmacon D-001910-01-05) were co-transfected; *YY1* siRNA (Dharmacon A-011796-16-0005) was transfected as a positive control. For gain-of-function, different amounts of pCDNA3-HA-*TCF7* (0, 30 or 100 ng, Addgene #40620) or pCDNA3-*CTNNB1* plasmids (0, 30, 100 ng, Addgene #16828) were co-transfected. As for GSK3β inhibitor treatments, 1 µM BIO or 10 µM SB216763 was added 12 h after transfection. Cells were collected 48 h later, and enhancer activity was determined using the Dual Luciferase Reporter Assay System (Promega, E1910) as described by the manufacturer’s protocol.

### ChIP-PCR assay

ChIP-PCR assays were performed on five million naive CD4 T cells isolated from healthy individuals using the ChIP-IT Kit (53040) from Active Motif. Oligonucleotide primers were designed to amplify pri-miR-181a/b1 sequences (chr1:198,904,100-169): forward 5ʹ- CTCATTGTCTTTCAGCACTTTAC-3ʹ, and reverse 5ʹ- GAAATCTAATGGCCCACAAAAATA-3ʹ; normal IgG was used as negative control. Genomic DNA from naive CD4 T cells was used as input control.

### Quantitative PCR

Total RNA was extracted with the RNeasy Plus Micro Kit (Qiagen, 74034) or TRIzol™ Reagent (ThermoFisher, 15596026), and cDNA was synthesized using the High-Capacity RNA-to cDNA Kit (Applied Biosystems, 4387406). qPCR was performed in duplicates in 384-well plates using the ABI 7900HT System with Taqman Universal Master Mix II (Thermo Fisher, 4440040) using the following probes: pri-miR-181a/b1 (Taqman, HS03302966), 18 S rRNA (Taqman, Hs9999) and *TCF7* (Taqman, HS01556515), or PowerUp SYBR Green Master Mix (ThermoFisher, A25742) using the following primers: *TCF7* (forward 5ʹ-AGAGAGAGTTGGGGGACACC-3ʹ, reverse 5ʹ-TACTCCGCCTTCAATCTGCT-3ʹ), β-actin (forward 5ʹ-GCACAGAGCCTCGCCTT-3ʹ, reverse 5ʹ-GTTGTCGACGACGAGCG-3ʹ). *PIM1* (forward 5ʹ- GGCTCGGTCTACTCAGGCA-3ʹ, reverse 5ʹ- GGAAATCCGGTCCTTCTCCAC-3ʹ)

### Flow cytometry

One million T cells were collected and washed with PBS containing 2% BSA and cells were stained with live/dead (Aqua) stain, anti-CD4, anti-CD45RA, and anti-CD62L antibodies for 30 min on ice. Cells were washed once and stained for TCF1 according to the manufacturer protocol (Biolegend Cat. No.424401). In general, cells were fixed in 1 mL fixation buffer for 10 min at room temperature, then 2 mL permeabilization (perm) buffer was added directly and cells were centrifuged for 5 min at 600 g. The supernatant was discarded and cells were resuspended in 1 mL of perm buffer and then pelleted and resuspended in 50 µL perm buffer and 2 µL Alexa Fluor-647 anti-TCF1(Biolegend:655204) was added and incubated for 45 min at room temperature. Cells were washed with 2 mL PBS containing 2% BSA once, and analyzed by flowcytometry.

### T cell functional assays

Naive CD4 T cells were treated with two different GSK3β inhibitors, BIO (1 µM) or SB216763 (5 µM), for 48 h. Treated cells were incubated with 1 µg/mL CD3/CD28 antibody for 30 mins on ice and cross-linked with anti-mouse IgG at indicated time points. ERK phosphorylation was measured by Western blot. To assess IL-2 production, cells were cultured with anti-CD3/CD28 Dynabeads (cell-to-bead ratio 2:1) for 24 h with GolgiPlug (BD Biosciences, 555029) added for the last 8 h. Cells were harvested, fixed, and permeabilized followed by staining with Alexa-Fluor-488-conjugated anti-human IL-2 antibodies, and then analyzed on a BD LSR Fortessa cytometer. To determine whether observed effects were mediated by increased miR-181a, naive CD4 T cells were transfected with miR-181a inhibitor or miRNA inhibitor negative control using the P3 Primary cell 4D-Nucleofector System (Lonza). Twenty-four hours later, cells were treated with GSK3β inhibitor BIO (1 µM) or SB216763 (5 µM) for 48 h. ERK phosphorylation and IL-2 production were assessed as described above. To assess CD69 and CD25 expression, cells were cultured in plates coated with anti-CD3/CD28 antibodies at 2 µg/mL for 24 h or 5 µg/mL for 48 h, respectively. For cell proliferation assay, cells were pretreated with CellTrace^TM^ violet (ThermoFisher scientific C34557) and seeded in plates coated with anti-CD3/CD28 antibodies at 5 µg/mL. Cell proliferation was analyzed by flow cytometry; dead cells were excluded on the basis of SYTOX^TM^ staining (ThermoFisher scientific s34860). Naive CD4 T cells were pretreated with 0.5 µM PIM1 inhibitor for 24 h, then cells were washed once and seeded on anti-CD3/CD28 coated plates for 24 and 48 h to assess expression of CD69 and CD25, respectively.

### Immunoblotting

Two million naive CD4 T cells from young or old healthy donors were treated with GSK3β inhibitors BIO (1 µM) or SB216763 (5 µM) for 48 h; in some experiments, cells were transfected with miR-181a inhibitor or miRNA inhibitor negative control before treatment. Cells were placed on ice and lysed with RIPA buffer (Thermo Scientific) in the presence of phosphatase and protease inhibitors, sodium orthovanadate, and PMSF (Santa Cruz Biotechnology). Approximately 10 µg of total protein was loaded in each well of SDS-PAGE gels and transferred to PVDF membranes. Membranes were incubated with primary antibodies specific for TCF1, DUSP6, and pERK, then HRP-labeled secondary antibody and developed with Pierce ECL Western blotting substrate (Thermo Fisher Scientific). Membranes were stripped with stripping buffer (Invitrogen) and the loading controls were re-probed with anti-β-actin antibodies.

### Statistical analysis

Statistical analysis was performed with Prism software (GraphPad) using paired or unpaired two-tailed t-tests or one-way analysis of variance (ANOVA) with post hoc Tukey test. Sample sizes in the population studies were chosen to ensure 80% power with α of 0.05 for detecting a difference of 1.0 standard deviation between populations. *P* < 0.05 was considered significant.

### Reporting Summary

Further information on research design is available in the Nature Research Reporting Summary linked to this article.

## Supplementary information

Supplementary Figures

reporting summary

## Data Availability

All data needed to evaluate the conclusions in the paper are present in the paper and/or the Supplementary information. Additional data available from authors upon request.
