# Supplementary material for: The GSK3β-β-catenin-TCF1 pathway improves naive T cell activation in old adults by upregulating miR-181a
Source: NPJ Aging Mech Dis. 2021 Feb 8;7:4. doi: 10.1038/s41514-021-00056-9 (PMC7870817; doi:10.1038/s41514-021-00056-9)
Supplement: Supplementary file 1 — Supplementary Figures [file 41514_2021_56_MOESM1_ESM.docx]

**Supplementary information**

**The GSK3β-β-catenin-TCF1 pathway improves naïve T cell activation in old adults by upregulating miR-181a**

Zhongde Ye, Timothy M. Gould, Huimin Zhang, Jun Jin, Cornelia M. Weyand, Jörg J. Goronzy


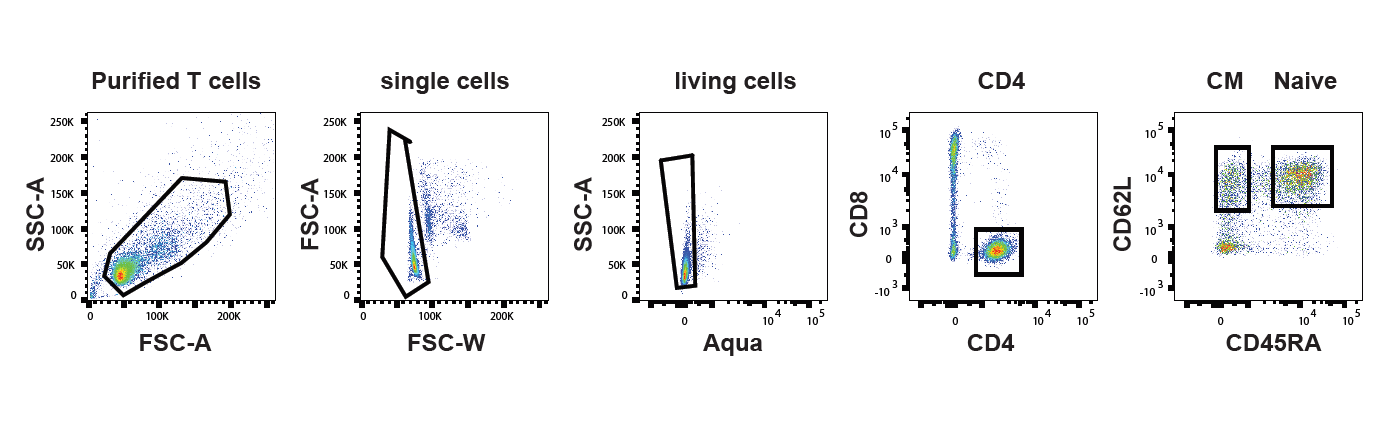


**Supplementary Figure 1. Flow cytometry gating strategy.**

Gating strategy for flow cytometry studies of naïve CD4 T cells, related to experiments in Fig. 2c on TCF1 expression and sorting of naïve or central memory CD4 T cells in supplementary Figures 2 and 3.


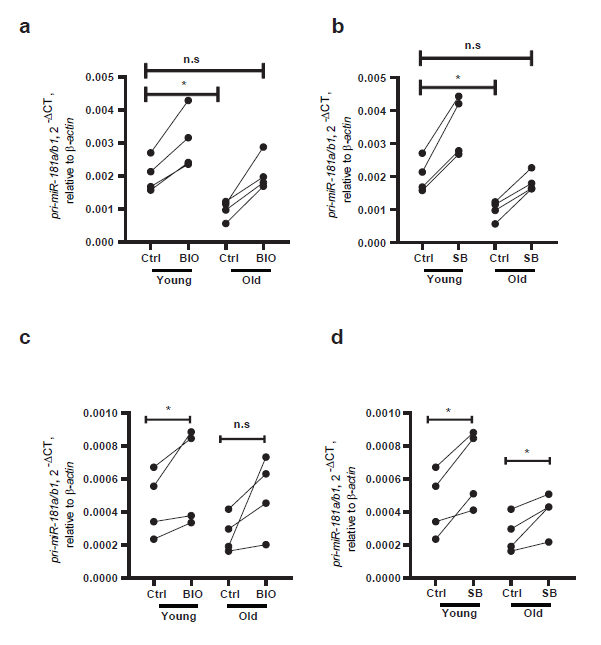


**Supplementary Figure 2**. **Upregulation of miR-181a expression in CD4 T cells by inhibition of GSK3ß**

(**a, b**) Naïve CD4 T cells (CD4^+^CD45RA^+^CD62L^+^) or (**c, d**) central memory CD4 T cells (CD4^+^CD45RA^-^CD62L^+^) from four young and four old individuals were purified by cell sorting cultured with DMSO solvent control or BIO (a, c, 1 µM) or SB216763 (b, d, 5 µM) for 48hrs. Pri-miR-181a/b1 transcripts were quantified by qPCR. Comparisons were done by two-tailed unpaired t test in a, b and paired t test in c, d. Significance levels are indicated as *P < 0.05, **P < 0.005, ***P < 0.0001.

**
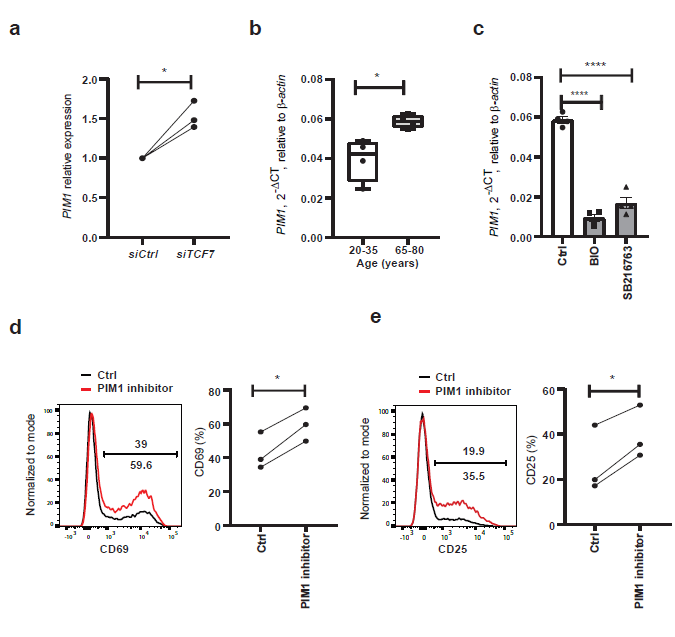
**

**Supplementary Figure 3. TCF1 silencing regulates TCR signaling through upregulating PIM1.** (a) Silencing of TCF7 upregulates PIM1 transcripts in naïve CD4 T cells (n=3). (**b**) Comparison of naïve CD4 T cells from young and old adults for PIM1 expression. (**c**) GSK3ß inhibition downregulates PIM1 expression in naïve CD4 T cells from old adults (n=4). (**d**) Inhibition of PIM1 increases CD3/CD28-activated expression of CD69 in naïve CD4 T cells from old adults. Representative histograms (left) and frequencies of CD69-positive cells are shown as mean ± SEM (n=3, right). (**e**) Inhibition of PIM1 increases CD3/CD28-activated expression of CD25 expression in naïve CD4 T cells from old adults. Representative histograms (left) and frequencies of CD25-expressing cells are shown as mean ± SEM (n=3, right). Comparisons were done by two-tailed unpaired (a, b) or paired (d, e) t tests; or by one-way ANOVA with post-hoc Tukey test in c. Significance levels are indicated as *P < 0.05, **P < 0.005, ****P < 0.00001.


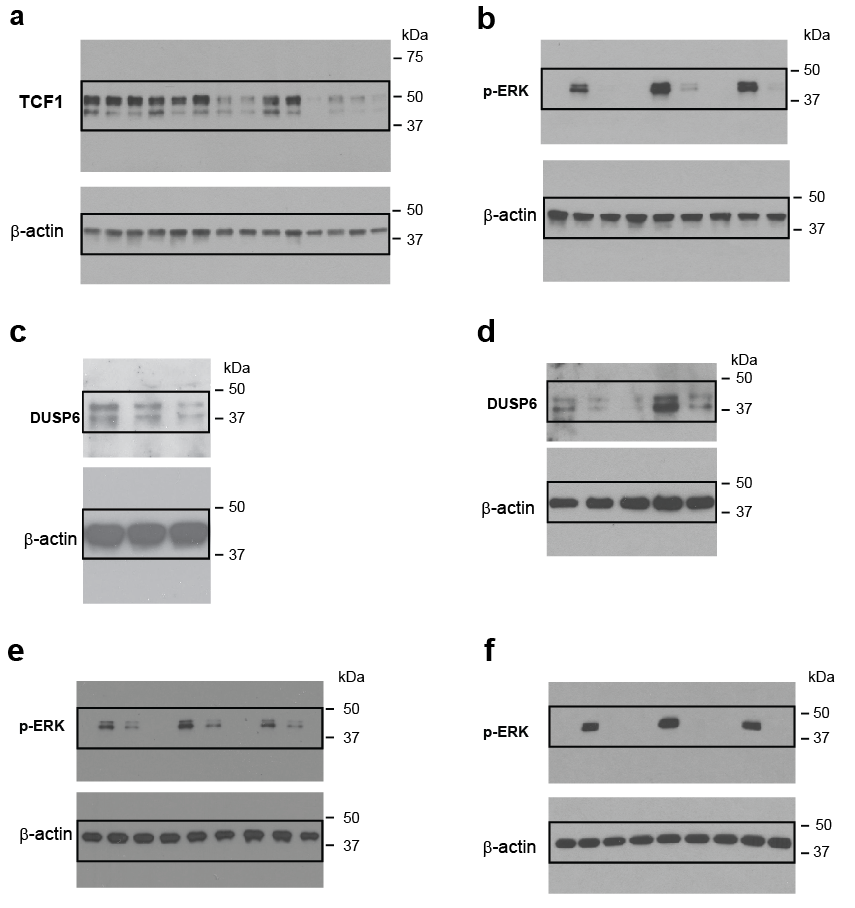


**Supplementary Figure 4.** **Original immunoblot corresponding to Fig. 2b (a), Fig. 3a (b), and Fig. 4a-d (c-f).**
