# Supplementary material for: The GSK3β-β-catenin-TCF1 pathway improves naive T cell activation in old adults by upregulating miR-181a
Source: NPJ Aging Mech Dis. 2021 Feb 8;7:4. doi: 10.1038/s41514-021-00056-9 (PMC7870817; doi:10.1038/s41514-021-00056-9)
Supplement: Supplementary file 2 — reporting summary [file 41514_2021_56_MOESM2_ESM.pdf]

nature research

Corresponding author(s): Jorg Goronzy, MD PhD

Last updated by author(s): Dec 16, 2020

Reporting Summary

Nature Research wishes to improve the reproducibility of the work that we publish. This form provides structure for consistency and transparency in reporting. For further information on Nature Research policies, see our Editorial Policies and the Editorial Policy Checklist.

Statistics

For all statistical analyses, confirm that the following items are present in the figure legend, table legend, main text, or Methods section.

n/a	Confirmed	
☐	☒	The exact sample size (*n*) for each experimental group/condition, given as a discrete number and unit of measurement
☐	☒	A statement on whether measurements were taken from distinct samples or whether the same sample was measured repeatedly
☐	☒	The statistical test(s) used AND whether they are one- or two-sided<br>*Only common tests should be described solely by name; describe more complex techniques in the Methods section.*
☒	☐	A description of all covariates tested
☐	☒	A description of any assumptions or corrections, such as tests of normality and adjustment for multiple comparisons
☐	☒	A full description of the statistical parameters including central tendency (e.g. means) or other basic estimates (e.g. regression coefficient) AND variation (e.g. standard deviation) or associated estimates of uncertainty (e.g. confidence intervals)
☐	☒	For null hypothesis testing, the test statistic (e.g. *F*, *t*, *r*) with confidence intervals, effect sizes, degrees of freedom and *P* value noted<br>*Give P values as exact values whenever suitable.*
☒	☐	For Bayesian analysis, information on the choice of priors and Markov chain Monte Carlo settings
☒	☐	For hierarchical and complex designs, identification of the appropriate level for tests and full reporting of outcomes
☒	☐	Estimates of effect sizes (e.g. Cohen's *d*, Pearson's *r*), indicating how they were calculated

Our web collection on statistics for biologists contains articles on many of the points above.

Software and code

Policy information about availability of computer code

Data collection	No software was used for data collection
Data analysis	PRISM 8.4 (GraphPad), FlowJo (Tree Star Inc).

For manuscripts utilizing custom algorithms or software that are central to the research but not yet described in published literature, software must be made available to editors and reviewers. We strongly encourage code deposition in a community repository (e.g. GitHub). See the Nature Research guidelines for submitting code & software for further information.

Data

Policy information about availability of data

All manuscripts must include a data availability statement. This statement should provide the following information, where applicable:
- Accession codes, unique identifiers, or web links for publicly available datasets
- A list of figures that have associated raw data
- A description of any restrictions on data availability

All data that support the findings in this study are available from Dr. Goronzy (corresponding author) upon reasonable request.

Field-specific reporting

Life sciences study design

All studies must disclose on these points even when the disclosure is negative.

Sample size	The sample size was calculated to ensure 80% power to detect a group difference of 2.0 standard deviation.
Data exclusions	No data were excluded.
Replication	Data were reproduced using various techniques such as qPCR, Western blotting and flow cytometry. All findings reported in this manuscript were reproducible.
Randomization	N/A
Blinding	Investigator at the time of measurement was blinded to the group assignment.

Reporting for specific materials, systems and methods

We require information from authors about some types of materials, experimental systems and methods used in many studies. Here, indicate whether each material, system or method listed is relevant to your study. If you are not sure if a list item applies to your research, read the appropriate section before selecting a response.

Materials & experimental systems

n/a	Involved in the study	
☐	☒	Antibodies
☒	☐	Eukaryotic cell lines
☒	☐	Palaeontology and archaeology
☒	☐	Animals and other organisms
☐	☒	Human research participants
☒	☐	Clinical data
☒	☐	Dual use research of concern

Methods

n/a	Involved in the study	
☒	☐	ChIP-seq
☐	☒	Flow cytometry
☒	☐	MRI-based neuroimaging

Antibodies

Antibodies used	All antibodies are commercially available and source information is provided in material and methods.
Validation	All antibodies have been validated by the respective company.

Human research participants

Policy information about studies involving human research participants

Population characteristics	Healthy adults of different ages, most of them blood donors
Recruitment	Samples were leftover samples from blood donors selected for age range. Few samples were collected from a healthy donor cohort maintained in our program.
Ethics oversight	Stanford University IRB

Note that full information on the approval of the study protocol must also be provided in the manuscript.

Flow Cytometry

Plots

Confirm that:

☒ The axis labels state the marker and fluorochrome used (e.g. CD4-FITC).

☒ The axis scales are clearly visible. Include numbers along axes only for bottom left plot of group (a 'group' is an analysis of identical markers).

☐ All plots are contour plots with outliers or pseudocolor plots.

☒ A numerical value for number of cells or percentage (with statistics) is provided.

Methodology

Sample preparation	Peripheral blood mononuclear cells were isolated by gradient centrifugation. T cell subpopulations were isolated from PBMC using Enrichment kits from STEMCELL Technologies as described in the Methods section, or by sorting with FACS sorter
Instrument	LSR II or Fortessa cytometer (BD Biosciences).
Software	FlowJo
Cell population abundance	Isolated cell populations were >95% pure.
Gating strategy	Naive CD4 T cells were gated as CD4+CD45RA+CD62L+, central memory CD4 T cells were gated as CD4+CD45RA-CD62L+. See supplementary Figure 1

☒ Tick this box to confirm that a figure exemplifying the gating strategy is provided in the Supplementary Information.
